# MRI-derived radiomics model for baseline prediction of prostate cancer progression on active surveillance

**DOI:** 10.1038/s41598-021-92341-6

**Published:** 2021-06-21

**Authors:** Nikita Sushentsev, Leonardo Rundo, Oleg Blyuss, Vincent J. Gnanapragasam, Evis Sala, Tristan Barrett

**Affiliations:** 1 Department of Radiology, Addenbrooke’s Hospital, University of Cambridge School of Clinical Medicine, Cambridge Biomedical Campus, Box 218, Cambridge, CB2 0QQ UK; 2 Cancer Research UK Cambridge Centre, University of Cambridge, Cambridge, UK; 3 School of Physics, Engineering & Computer Science, University of Hertfordshire, Hatfield, UK; 4 Department of Paediatrics and Paediatric Infectious Diseases, Sechenov First Moscow State Medical University, Moscow, Russia; 5 Department of Applied Mathematics, Lobachevsky State University of Nizhny Novgorod, Nizhny Novgorod, Russia; 6 Division of Urology, Department of Surgery, University of Cambridge, Cambridge, UK; 7 Cambridge Urology Translational Research and Clinical Trials Office, University of Cambridge, Cambridge, UK

**Keywords:** Cancer imaging, Urological cancer, Prostate

## Abstract

Nearly half of patients with prostate cancer (PCa) harbour low- or intermediate-risk disease considered suitable for active surveillance (AS). However, up to 44% of patients discontinue AS within the first five years, highlighting the unmet clinical need for robust baseline risk-stratification tools that enable timely and accurate prediction of tumour progression. In this proof-of-concept study, we sought to investigate the added value of MRI-derived radiomic features to standard-of-care clinical parameters for improving baseline prediction of PCa progression in AS patients. Tumour T_2_-weighted imaging (T2WI) and apparent diffusion coefficient radiomic features were extracted, with rigorous calibration and pre-processing methods applied to select the most robust features for predictive modelling. Following leave-one-out cross-validation, the addition of T2WI-derived radiomic features to clinical variables alone improved the area under the ROC curve for predicting progression from 0.61 (95% confidence interval [CI] 0.481–0.743) to 0.75 (95% CI 0.64–0.86). These exploratory findings demonstrate the potential benefit of MRI-derived radiomics to add incremental benefit to clinical data only models in the baseline prediction of PCa progression on AS, paving the way for future multicentre studies validating the proposed model and evaluating its impact on clinical outcomes.

## Introduction

Prostate cancer (PCa) is the second commonest and the sixth deadliest male cancer worldwide, with its incidence expected to double by 2030 due to an ageing male population^1,2^. In the UK, 43% of men present with low- and favourable intermediate-risk localised disease, for which level 1 evidence suggests non-inferiority of active surveillance (AS) to radical treatment in terms of 10-year survival^3–7^. However, the lack of consensus on both the stringency of inclusion criteria and the definition of disease progression have led to a significant variability of how AS protocols are run both across centres^8^ and indeed across guidelines^9^. As a result, a cumulative five-year dropout rate on AS reaches 44%, of which 27% are triggered by disease progression^10,11^. This highlights the unmet clinical need for developing robust baseline risk-stratification tools enabling early detection of patients harbouring lesions with a high potential for progression^12^. These patients would either require stricter AS follow-up or should be considered for radical treatment.

Numerous studies recently summarised in a systematic review by Sierra et al.^13^ have investigated the utility of both nomograms and individual clinicopathological predictors of histopathological progression in AS cohorts. However, none of the investigated models has entered routine clinical practice due to their low predictive accuracy and poor performance with external validation. One possible explanation for this may be the intrinsic difficulty of standardising the predictors, with an obvious example being PSA density that varies considerably depending on the imaging modality used for measuring prostate volume^14^. Furthermore, pathology results from needle biopsy may differ from the final pathology at prostatectomy due to sampling error, inter-observer variation, borderline grades, or upgrading due to oversampling the most radiologically suspicious area^10^. Conversely, the ability of MRI to visualise the whole tumour volume coupled with ongoing attempts to standardise image acquisition parameters^15^ provide the potential to investigate the ability of quantitative image-derived features, or radiomics, to advance the development of accurate and reproducible predictors of disease progression on AS.

In PCa, a considerable body of radiomics research has focused on improving the detection of clinically significant disease^16–18^ to address the moderate positive predictive value of qualitative mpMRI assessment and reduce the overdiagnosis of indolent disease^19,20^. Zhang et al.^21^ recently introduced a radiomic model predicting histopathological upgrading of PCa from biopsy to radical prostatectomy, which may also improve baseline lesion characterisation. Furthermore, radiomics models have been developed to preoperatively predict the probability of extracapsular extension^22–24^, which is essential for accurate local staging of the disease and subsequent clinical decision-making. However, to the best of our knowledge, no attempts have been made to utilise radiomics for predicting disease progression in patients enrolled on AS programmes, the proportion of which is growing steadily in North America, Europe, and Australia^25^.

Therefore, in this proof-of-concept study, we sought to develop a radiomics model that would predict the baseline risk of PCa progression on AS. To achieve this, we extracted MRI-derived intratumoural radiomic features from AS patients with and without disease progression over a similar follow-up period. The predictive performance of the resulting sequence-specific radiomic models was assessed both individually and in combination with a clinical predictive model consisting of standard-of-care baseline clinicopathological biomarkers, with the aim of identifying a combined model with the highest overall performance to inform the future work in the field.

## Methods

### Patient population

This retrospective case–control study was part of a service evaluation of the prostate diagnostic pathway, with the need for informed consent for data analysis waived by the local institutional review board (NRES Committee East of England, UK). All experimental protocols of this study were carried out in accordance with the Declaration of Helsinki, as well as all the relevant guidelines and regulations, and were approved by the aforementioned local institutional review board. The study included patients with biopsy-proven PCa visible on both T_2_-weighted Imaging (T2WI) and apparent diffusion coefficient (ADC) maps, who were enrolled on active surveillance according to local criteria^26^ and had a minimum follow-up of two years with at least two consecutive 3 T MRI scans performed on the same magnet. The exclusion criteria were patients on AS with no MR-visible lesion, undergoing any prior treatment for PCa or benign disease, or the presence of total hip replacement or other pelvic metalwork.

Patients were divided into two groups depending on disease progression status. Cases were represented by patients who demonstrated disease progression, which was defined as a switch to radical treatment triggered by either confirmed histopathological progression on repeat targeted biopsy, or definitive radiological stage progression of the target lesion (MRI-derived PRECISE^23^ score 5). The control group included patients in whom the disease was regarded stable over the same follow-up period, with no signs of radiological progression recorded on all prospective MRI scans (PRECISE score 3) and no histopathological progression noted on all repeat targeted biopsies, with both criteria being compulsory.

### Baseline clinicopathological predictors of disease progression

To investigate the predictive value of standard-of-care clinicopathological predictors of disease progression, the following baseline parameters were collected for all included patients: PSA, MRI-derived gland volume, PSA density, MRI-derived Likert score of tumour probability, target lesion localisation (peripheral zone or transition zone), and target lesion biopsy grade group.

### Biopsy technique

Depending on clinical recommendation, biopsy was performed by either a transrectal or transperineal approach, using MRI/ultrasound fusion. All biopsy procedures were performed by experienced urologists and included 12–24 systematic cores, with 2–4 separate target cores acquired from the MRI defined lesion/s. All targets were defined by radiologists pre-procedure using T2WI as the primary and diffusion-weighted imaging as the secondary source images, using the DynaCAD system (InVivo Corp, Orlando, FL, USA) for transrectal and Biopsee software (Oncology Systems Limited, Shrewsbury, UK) for transperineal approaches as previously described^24^.

### MRI acquisition parameters

Patients underwent prostate MRI on a 3 T MR750 scanner (GE Healthcare, Waukesha, WI) using a 32-channel receiver coil. Intravenous injection of hyoscine butylbromide (Buscopan, 20 mg/mL; Boehringer, Ingelheim am Rhein, Germany) was administered prior to imaging to reduce peristaltic movement, unless clinically contraindicated. Multiparametric MRI protocol included Axial T_1_ and multiplanar high-resolution T_2_-weighted 2D fast recovery FSE (field of view (FOV) 18 × 18 cm^2^; voxel size 0.35 × 0.35 mm^2^; slice thickness 3 mm; gap 0 mm). Diffusion-weighted imaging (DWI) was performed using a spin-echo echo-planar imaging pulse sequence (FOV 28 cm; slice thickness 3 mm; gap 0 mm; b-values: b-150, b-750, and b-1,400 s/mm^2^) and an additional small FOV (24 cm) b-2,000 s/mm^2^ DWI sequence; ADC maps were calculated automatically. Dynamic contrast enhancement was performed using a standard sequence (FOV 24 cm; slice thickness and gap 3 mm and 0 mm, respectively; temporal resolution 7 s) following a bolus of Gadobutrol (Gadovist, 0.1 mmol/kg; Bayer, Leverkusen, Germany) at 28 s via a power injector, at a rate of 3 mL/s (dose 0.1 mmol/kg).

### Image analysis and segmentation

The MR images were interpreted prospectively by one of four sub-specialist uro-radiologists with 5–16 years’ experience of reporting prostate MRI, with each having read > 2,000 cases to be considered experts^27,28^. MRI sequences were evaluated based on the Prostate Imaging-Reporting and Data System (PI-RADS) structured scoring criteria^29^. An overall impression was then used to derive a Likert suspicion score, wherein Likert 1 = clinically significant prostate cancer (csPCa) highly unlikely, 2 = csPCa unlikely, 3 = indeterminate for csPCa, 4 = csPCa likely, 5 = csPCa highly likely^30^. The assigned Likert scores were subsequently reviewed by independent readers as part of multidisciplinary team meetings.

**Figure 1:**
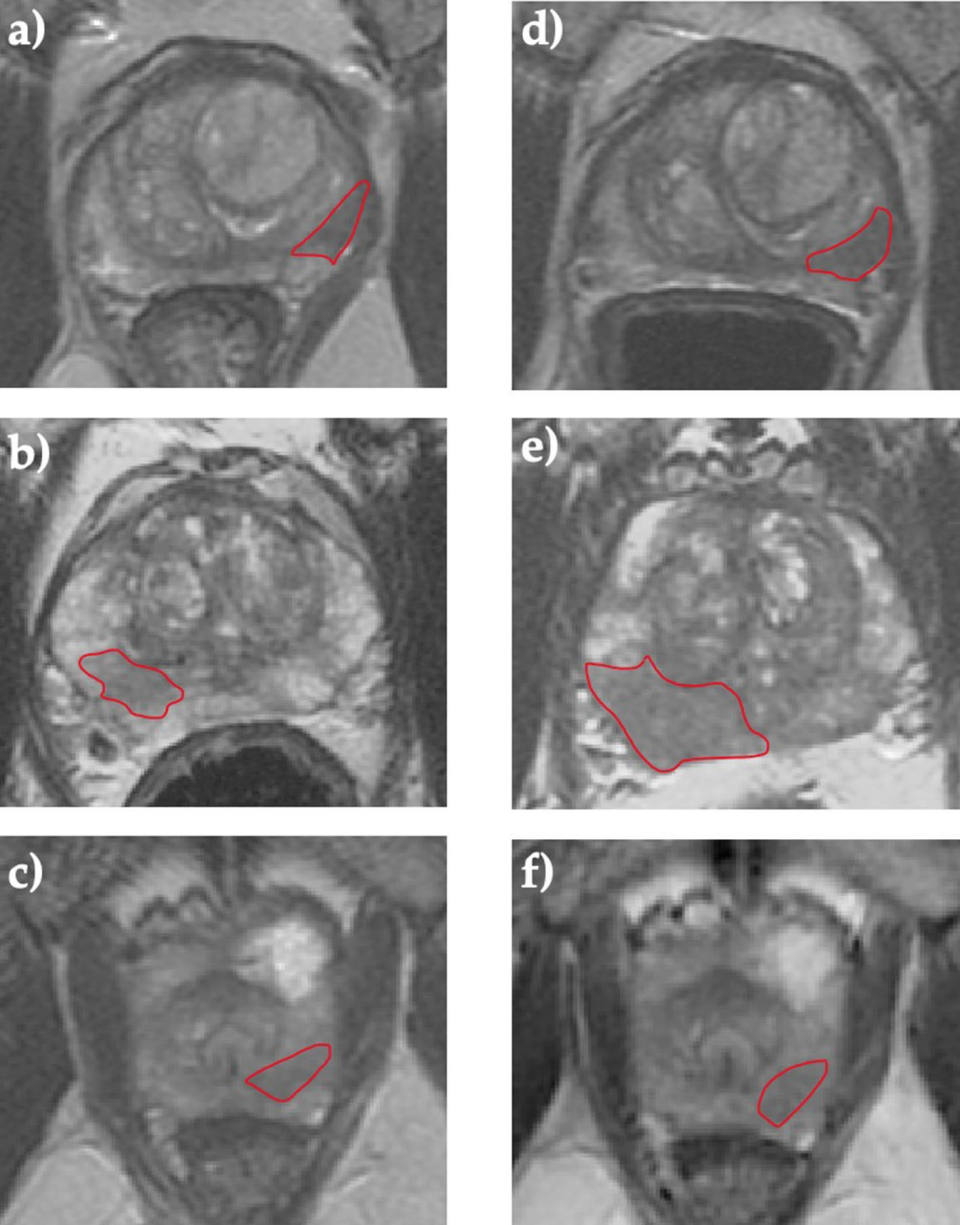
Comparison of T2-weighted images of the prostate obtained at baseline pre-biopsy (**a**–**c**) and follow-up (**d**–**f**) mpMRI scans from patients enrolled on active surveillance. Images (**a**,**d**) were obtained from a patient with stable 3 + 3 = 6 disease that showed neither radiological not histopathological progression over a follow-up period of three years. Images (**b**,**e**) were obtained from a patient with both radiological (PRECISE 4) and histopathological (3 + 4 = 7 to 4 + 3 = 7) progression. Images (**c**,**f**) were obtained from a patient with confirmed histopathological progression (3 + 3 = 6 to 3 + 4 = 7) but radiologically stable disease (PRECISE 3). The best-performing predictive model (T2WI-derived radiomic features, PSA and PSA density) predicted the clinical outcome in all three presented cases.

For texture analysis, tumour ROIs were drawn on anatomical T2WI (example ROIs are presented in Fig. 1) and ADC maps by a fellowship-trained uro-radiologist with 12 years’ experience of reporting prostate MRI (TB) and an imaging research fellow (NS). The segmentation was performed in consensus using the open-source segmentation software ITK-SNAP^25^. The reliability of image segmentation was evaluated by applying ROI morphological opening and closing using the SciPy Python package.

### Radiomics analysis

**Figure 2:**
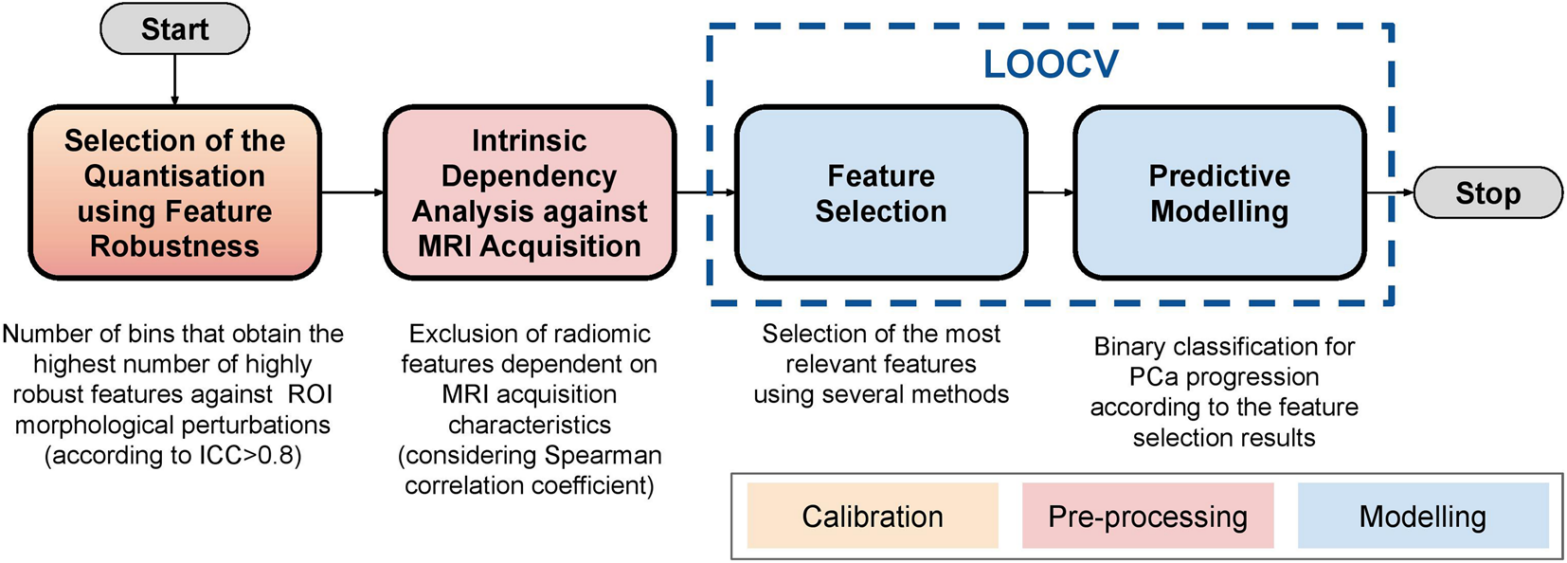
Overall workflow of the radiomics pipeline used in this study. The main phases are: (i) calibration, (ii) pre-processing, and (iii) predictive modelling with leave-one-out cross validation (LOOCV), for which a colour legend is shown in the bottom right corner.

Figure 2 illustrates the overall workflow of the radiomics pipeline utilised to develop and validate predictive models for PCa progression on AS. The key stages of the pipeline are described in the following sections.

### Radiomic feature extraction

The analysed features were extracted using PyRadiomics^31^, which is an open-source Python package developed for the standardisation of radiomic feature extraction^32^. We used PyRadiomics version 2.0 and Python 3.7.5. Six feature classes were extracted: (1) first-order intensity histogram statistics, (2) Grey Level Co-occurrence Matrix features (GLCM)^33,34^, (3) Grey Level Run Length Matrix (GLRLM)^35^, (4) Grey Level Size Zone Matrix (GLSZM)^36^, (5) Grey Level Dependence Matrix (GLDM)^37^, and (6) Neighbouring Gray Tone Difference Matrix (NGTDM)^38^. All extracted features are listed in Supplementary Table S1.

3D feature computation without any resampling was used to avoid interpolation artifacts. According to the Initiative for Biomarker Standardization Initiative (IBSI)^32^, the use of the number of bins is favoured over the bin width in the case of arbitrary intensity units, such as MRI. For the same motivation, no resegmentation (i.e. the voxels outside a specified range are removed from the mask prior to texture feature calculation) was applied. More details are available in the Supplementary Methods.

### Calibration and pre-processing

We aimed at reducing the initial set of extracted features by a subset of features that are both robust against ROI perturbations and independent from the MRI acquisition parameters.

Firstly, with the goal of identifying the robust features, we perturbed the original prostate cancer ROIs by using morphological operators (opening and closing with a 3D spherical structuring element of 1-pixel radius)^39^. By so doing, we produced three versions for each ROI (namely, original, opening and closing). This procedure simulates the variability of the ROIs, by considering intra- and inter-reader dependence during manual contouring^40^. Starting from these three sets of ROIs, the radiomic features were extracted separately for T2WI and ADC by using different quantisation configurations: the number of bins varied in {8, 16, 32, 64, 128, 256}. Thus, the two perturbations applied to the original ROIs and the different quantization settings yielded 18 configurations of radiomic features. Therefore, the ROI perturbations were aimed at assessing the most robust quantisation setting (i.e. rebinning).

The ICC was considered to determine the most robust features against the ROI perturbations whilst the number of bins varied too^41^. ICC analysis was applied to these 18 configurations of features to establish the number of bins that achieved the largest set of robust features for T2WI and ADC separately. In particular, we considered the two-way random-effects model (or mixed-effects), consistency, single rater/measurement, ICC(3,1)^42^:

1 $$ {\text{ICC}}\left( {3,1} \right) = \frac{{MS_{R}  - ~MS_{E} }}{{MS_{R} ~ + ~\left( {k - 1} \right)~MS_{E} }}, $$

where ${MS}_{R}$, ${MS}_{E}$ and ${MS}_{C}$ are the mean square for rows, mean square for error and mean square for columns, respectively*.*

The chosen number of bins represents the most reliable quantisation configuration (i.e. rebinning) according to the ROI perturbations via morphological operators. A cut-off value of 0.8 was used for the ICC to identify the number of features with high robustness. The used quantisation configuration was selected accordingly, by considering the number of bins that obtained the highest number of robust features for both T2w and ADC. The highly robust features with ICC > 0.8 were then used in the downstream pre-processing phases. ICC > 0.9 is the setting used for ‘excellent robustness’, however, in our predictive radiomics analysis we chose the less aggressive cut-off ICC > 0.8 as a measure for ‘high robustness’, representing a compromise between ICC > 0.9 and ‘high or moderate robustness’ (ICC > 0.5)^41,43^.

Secondly, the extracted features might be affected by the MRI acquisition characteristics, such as scanner type, scanner settings, imaging protocols and acquisition parameters^44^. In particular, we calculated the Spearman correlation coefficient for each radiomic feature against each considered MRI acquisition parameter, namely: (i) echo time (TE); (ii) repetition time (TR); (iii) flip angle; (iv) slice thickness; (v) spacing between slices; (vi) pixel spacing. A test–retest analysis for assessing feature robustness was not performed given that clinical MRI scans were used^32^. Our aim was to decouple possible dependencies between each feature and the principal MRI acquisition parameters in our highly homogeneous dataset, thus, this pre-processing step was based just on a simple Spearman’s correlation.

### Feature selection and predictive modelling

In order to accommodate the high-dimensional nature of radiomics, minimise potential model overfitting, and reduce the dimensionality, we used six feature selection approaches including Fisher Score (FSCR), T-Score (TSCR), Wilcoxon Score (WLCX), Gini index (Gini), Multivariate Mutual Information Maximization (MIM), and Minimum Redundancy Maximum Relevance (MRMR). In this proof-of-concept study, we did not intend to build a single best-performing model—instead, we aimed to investigate whether the radiomic features were complementary to clinical features. Therefore, we limited the total number of features selected by commonly applied cut-offs. Given the low number of available clinical features, we used the top 5 features from each approach, whereas for each radiological dataset, the total number of features was restricted to 10, leading us to incrementally use the top 15 features for each combination.

Following the identification of the optimal number of features, several machine learning techniques were developed to incorporate these features into a binary classifier for distinguishing patients with the disease progression from those who remained stable over the study period. Methods used included k-nearest neighbours (KNN), logistic regression (LG), linear discrimination analysis (LDA), general linear model (GLMnet), support vector machine (SVM) and random forest (RF), with the analysis settings set as default. Given a limited sample size of the data, a leave-one-out cross-validation (LOOCV) scheme was used in order to further minimise the risk of overfitting. In brief, to investigate the predictive ability of clinical and radiomic features, every machine learning approach was trained using data from all the patients but one, with all the hyperparameters tuned and then applied to the remaining patient. The default set of hyperparameters was used, including *k* and *λ* in the KNN and GMLnet methods, respectively, which were tuned independently at each step of the LOOCV procedure. This approach enabled us to demonstrate the overall predictive ability of the analysed dataset rather than developing one best-performing model. Given the exploratory design, we did not plot the receiver operating characteristic (ROC) curves as they would be based on a LOOCV scheme that implies constructing and evaluating a number of models (as many as the number of patients included) rather than a single model.

### Statistical and computational analysis

Along with the PyRadiomics-based feature extraction, the pre-processing and calibration steps in the radiomic pipeline were performed using the MatLab® R2019b (64-bit version) environment (The MathWorks, Natick, MA, USA), whilst the feature selection and predictive modelling were performed in R 4.0.2 using the praznik, MXM, and caret packages.

## Results

### Patient characteristics

This case–control study included 71 patients followed-up on the AS programme in our centre between May 2013 and April 2020. 73 MR-visible lesions were included in the analysis, of which 35 showed confirmed disease progression (histopathological = 25; radiological = 10) and 38 remained stable over the study period; sample cases representing the two groups are presented in Fig. 1. 12/73 (16%), 29/73 (40%), and 32/73 (44%) of lesions harboured Likert scores 3, 4, and 5, respectively. The mean age of the study population was 65.3 ± 6.4 years, with no inter-group difference observed between the progressors and non-progressors (66.4 ± 6.1 years vs 64.2 ± 6.5 years, respectively; p = 0.099). The mean follow-up length in this study was 4.4 ± 2.2 years for non-progressors. The mean time to progression period for progressors was 4.1 ± 2.2 years vs 4.8 ± 2.9 years of follow-up for non-progressors (p = 0.107). Table 1 presents the inter-group comparison of the baseline clinicopathological predictors of the disease progression, with PSA and PSA density being significantly higher in progressors compared to non-progressors (p = 0.021 and 0.003, respectively).

**Table 1:** Intergroup comparison of standard-of-care baseline clinicopathological predictors of prostate cancer progression in patients enrolled on active surveillance.

Baseline predictor	Progressors (n = 34)	Non-progressors (n = 37)	p-value
	Median (IQR)		
PSA, ng/mL	7.0 (4.9–8.7)	4.9 (3.4–6.8)	0.021
Gland-volume, mL	45.0 (32.0–53.0)	44.8 (35.8–66.5)	0.329
PSA density	0.17 (0.10–0.22)	0.09 (0.06–0.15)	0.003
Likert score	4.0 (4.0–5.0)	4.0 (4.0–5.0)	0.532
Biopsy grade group 1 (3 + 3 = 6)	26	28	–
Biopsy grade group 2 (3 + 4 = 7)	8	10	
Target lesion in the peripheral zone	23	29	–
Target lesion in the transition zone	12	9	

### Radiomics analysis: calibration and pre-processing

Since the choice of the quantisation, or rebinning, is critical, we performed the robustness analysis based on the Intraclass Correlation Coefficient (ICC) computed on the features extracted from the original regions-of-interest (ROIs) and their perturbations using morphological operations (see “Methods”). The number of bins varied in {8, 16, 32, 64, 128, 256}, and the number of high robust features was identified by an ICC > 0.8. For a homogenous setting suitable for both T_2_-weighted imaging (T2WI) and apparent diffusion coefficient (ADC), we took into account the sum of the highly robust features for both sequences. Table 2 shows that 128 bins provided the most suitable quantisation configuration for T2w and ADC. Therefore, out of 107 features extracted by PyRadiomics, at 128 bins, the number of highly robust features was 34 and 59 for T2WI and ADC, respectively. The dependence analysis between the resulting radiomic features and MRI acquisition parameters—based on a Spearman correlation analysis—showed no interdependent features (p < 0.001), thus confirming the homogeneity of the analysed dataset in terms of MRI acquisition parameters.

**Table 2:** Number of features with high robustness (ICC > 0.8) by varying the number of bins in the quantisation step for radiomic feature extraction for T2w and ADC MR images separately.

Number of bins	Number of features with excellent robustness		
	T2WI	ADC	Combined
8	38	47	85
16	36	49	85
32	33	53	86
64	35	55	90
128	34	59	93
256	33	56	89

### Feature selection and predictive modelling

**Figure 3:**
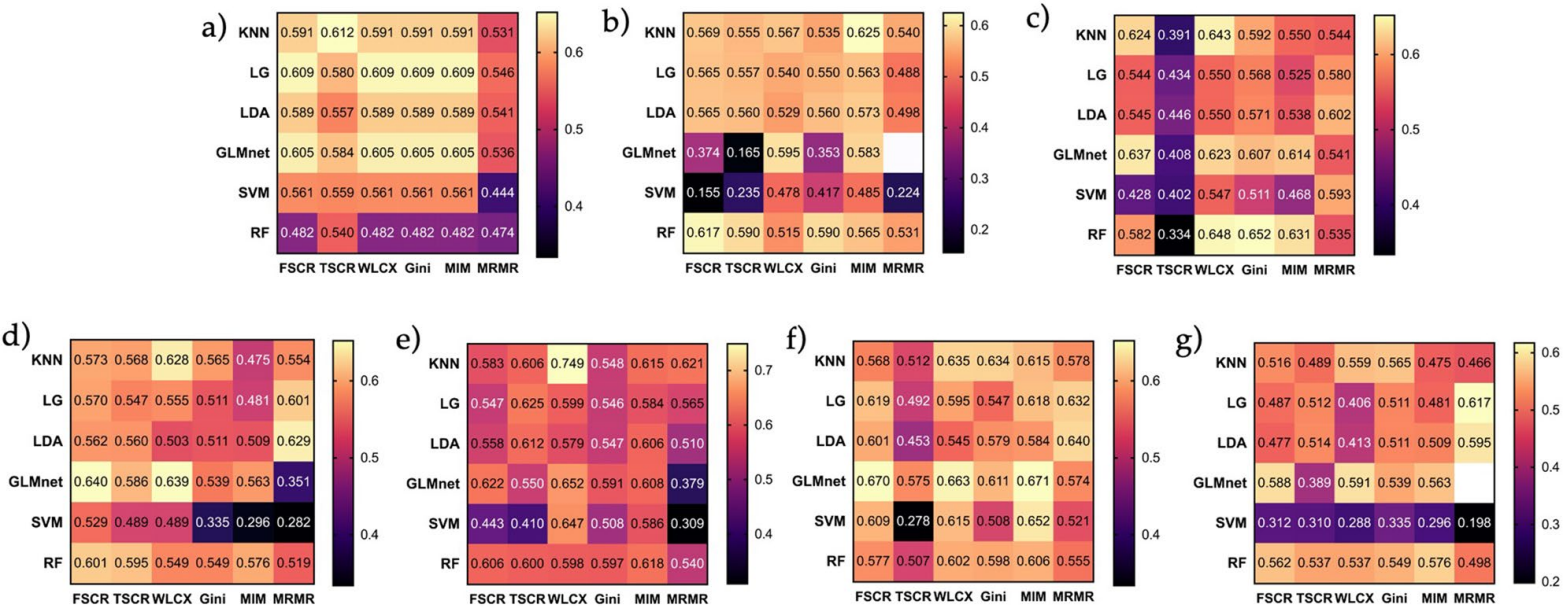
Heatmaps summarizing areas under the ROC curve (AUC) of predictive models developed including (**a**) clinicopathological predictors alone, (**b**) T2WI-derived radiomic features alone, (**c**) ADC-derived radiomic features alone, (**d**) a combination of clinicopathological predictors, T2WI-, and ADC-derived radiomic features, (**e**) a combination of clinicopathological predictors and T2WI-derived radiomic features, (**f**) a combination clinicopathological predictors and ADC-derived radiomic features, and (**g**) a combination of T2WI- and ADC-derived radiomic features. Each cell presents an AUC for a model developed using a given combination of feature selection and machine learning algorithms. 95% confidence intervals for each model are summarised in Supplementary Tables S1-7, respectively. Blank cells (**b**,**g**) denote models shrunk to the intercept only due to the regularisation approach used by GLMnet.

The areas under the ROC curve (AUCs) for clinicopathological predictors, T2WI-derived radiomic features and ADC-derived radiomic features alone, as well as for their combinations, are presented in Fig. 3, with 95% confidence intervals (CI) for each of the resulting models validated using LOOCV listed in Supplementary Tables S2-8**.** In order to account for the different numbers of clinical and radiomic features, we used the top 5 features for analysis of the clinical data, the top 10 features for each of the radiological data and top 15 features for combinations of these. Since GLMnet used a regularisation approach, there were two instances when the model shrunk to the intercept only and therefore was not reported (Supplementary Tables S3 and S8). The best predictive performance—AUC = 0.75 (95% CI 0.636–0.862)—was achieved for a model combining clinicopathological predictors with T2WI-derived radiomic features (Table 3) developed using the Wilcoxon signed rank test and k-nearest neighbours as feature selection and classification algorithms, respectively. Summary performance characteristics of the model based on sensitivity and specificity cut-offs are presented in Table 4. From a clinical standpoint, optimal performance is achieved with a combination of specificity 0.80 and sensitivity of 0.63. An intergroup comparison of the constituent features between progressors and non-progressors using the Wilcoxon rank sum test with Bonferroni correction revealed that only PSA-density was significantly different between the two groups (p = 0.03); Supplementary Fig. S1.

**Table 3:** Summary clinicopathological and T2WI-derived radiomic features comprising the best-performing predictive model of prostate cancer progression on active surveillance developed using the Wilcoxon signed rank test and k-nearest neighbours algorithms for feature selection and classification, respectively.

Feature class	Feature name
Clinicopathological predictor	PSA
	PSA density
Shape-based (3D)	Maximum 2D diameter (Row)
	Minor axis length
	Surface area
	Mesh volume
	Voxel volume
Grey level co-occurrence matrix (GLCM)	Informational measure of correlation 1
	Informational measure of correlation 2
	MCC: maximal correlation coefficient
Grey level run length matrix (GLRLM)	Grey level NonUniformity
	Run length NonUniformity
Grey level size zone matrix (GLSZM)	Grey level NonUniformity
Grey level dependence matrix (GLDM)	Grey level NonUniformity
Neighbouring grey-tone difference matrix (NGTDM)	Busyness

**Table 4:** Summary performance characteristics of the best-performing model (T2WI-derived radiomic features, PSA, and PSA density) from the leave-one-out cross validation results presented in Table 3.

Parameter	Specificity	Sensitivity	Sensitivity	Specificity
Combination	0.70	0.70	0.70	0.70
	0.75	0.67	0.75	0.63
	0.80	0.63	0.80	0.52
	0.85	0.54	0.85	0.34
	0.90	0.37	0.90	0.28

Depending on the clinical need, the predictive performance can be adjusted by prioritising specificity over sensitivity and vice versa, with the resulting parameter combinations presented in the table.

## Discussion

This proof-of-concept study investigates the added value of MRI-derived radiomics to standard-of-care clinicopathological biomarkers for baseline prediction of PCa progression on active surveillance in patients with MR-visible lesions. The combined predictive model, incorporating PSA and PSA density (PSAD) with selected T2WI-derived radiomic features, showed improved performance compared to models utilising clinical data alone. These results suggest a role for MRI-derived radiomics as an additional risk-stratification tool to triage patients suitable for AS depending on the progressive potential of the target lesions.

The role of mpMRI in identifying patients suitable for AS has been expanding gradually over the recent years, primarily building on its high negative predictive value for the presence of clinically significant disease unsuitable for AS^45^. Several studies have confirmed the ability of mpMRI to better identify men at risk of immediate reclassification at their initial assessment^46^, also highlighting the fact that the presence of an MR-visible lesion at baseline is associated with higher risk of disease progression on AS^12,47–52^. However, no systematic attempts other than in a diagnostic meta-analysis by Zhai et al*.*^53^ have been made to explore the ability mpMRI to further stratify MR-visible lesions based on their progressive potential in patients otherwise suitable for AS. Adding this extra risk-stratification element at baseline, which showed promise in other tumour types such as bladder^54^ and ovarian^55^ cancers, may improve clinical decision-making and help refine and personalise follow-up protocols. For these purposes, MRI-derived radiomics presents a promising approach due to its quantitative nature and the rapid development of novel machine learning algorithms for feature selection that may help overcome the known poor cross-system reproducibility of the technique^56,57^.

In this study, only two clinicopathological predictors (PSA and PSAD) were included in the best-performing model. These results are in line with those of previous studies, in which baseline PSAD had a significant effect on mean progression-free survival time in patients on AS programmes^12,52,58,59^. It should be noted that in our cohort, PSAD was largely determined by PSA rather than the gland volume, with the latter being broadly similar in both progressors and non-progressors, thereby explaining the inclusion of PSA in the best-performing model alongside PSAD. Given the relative homogeneity of the AS cohort in our centre, the inclusion of MR-visible lesions only (Likert scores 3–5), and the case–control nature of the study, other clinicopathological predictors (Likert score, biopsy ISUP grade group, and zonal location of lesions) showed no intergroup difference, thereby providing a possible explanation for the observed moderate performance of clinicopathological predictive models.

Interestingly, a similarly moderate predictive performance was demonstrated by both standalone and combined T2WI- and ADC-derived radiomic models. The moderate performance of ADC-derived radiomics may be explained by the intrinsically limited ability of ADC mapping to differentiate low- vs intermediate-risk disease using a clinical monoexponential-fit model, for which more advanced diffusion techniques such as diffusion kurtosis imaging (DKI) and Vascular, Extracellular, and Restricted Diffusion for Cytometry in Tumors (VERDICT) MRI have shown greater promise^60,61^. A considerable increase in the predictive performance was, however, achieved by a model combining T2WI-derived radiomic features with PSA and PSAD, highlighting a synergy between radiomic and clinicopathological features for improving baseline detection of lesions with high progressive potential. Of thirteen radiomic features included in the best-performing model, five were representative of the tumour shape, size, and volume, which aligns well with the results of previous studies suggesting a link between baseline MRI-derived lesion size and the disease progression on AS^52^. In terms of the model performance characteristics, the achieved specificity and sensitivity of 0.80 and 0.63 could be employed to focus its clinical use on identifying patients at low risk of progression who may benefit from less stringent AS protocols, maximising specificity whilst not being overly restrictive on patient uptake of AS. In addition to providing an evidence base for avoiding unnecessary biopsies, similar models might increase confidence of both patients and clinicians in the AS protocol chosen based on the low overall prostate cancer-specific mortality in AS cohorts even in patients with unfavourable intermediate-risk disease^62^.

This study has several limitations. Data were collected from a single centre, and additional external validation of the proposed model is required. This was, however, mitigated by the rigorous patient selection and matching, calibration, and pre-processing stages aimed at reducing overfitting and maximising the reliability of subsequent predictive modelling and cross-validation steps. Only patients with MR-visible lesions were included in the analysis, limiting generalisability of the findings, given that only 50% of patients included on AS programmes will have an MRI lesion detected^52,63^. The choice of such a cohort was, however, deliberate given that the presence of MR-visible lesions is in itself a poor prognosticator of PCa progression on AS, thereby warranting further risk-stratification of lesions based on their progressive potential. Dynamic contrast-enhanced images, obtained as part of mpMRI protocol, were not used in this study due to the increasing body of evidence suggesting the non-inferiority of biparametric MRI for lesion characterisation^64,65^. Furthermore, in this study we used Likert scores, which is the default system used in our department being informed by the criteria defined in PI-RADS v2.1. The systems are broadly similar, but Likert will often differ in scores 4 and 5 as it lacks a size threshold for this differentiation. The non-inferiority of Likert score assessment for the detection of clinically significant disease was documented previously^64,66^.

In future work, we will aim to deploy a whole-gland segmentation approach, alongside habitat radiomics, to develop similar predictive models for use in all patients eligible for AS regardless of the presence of MR-visible lesions. The increased sample size will allow us to identify the best-performing machine learning technique that uses the optimal total number of features from each relevant feature selection approach. To assess its performance, we will use conventional measures of discrimination and calibration and perform decision curve analysis. A posteriori statistical harmonisation, such as the ComBat method^67^, for explicitly dealing with the batch-effect correct in multicentric studies will be used in our future work. In such a context, automated prostate segmentation approaches^68,69^ can accelerate the outlining time in manual segmentation procedures, as well as reduce the operator dependence for repeatable radiomic feature extraction. In addition, prostate zonal segmentation^70,71^ might also be considered for extracting zone-specific radiomics biomarkers. Furthermore, as the intrinsic inconsistency of weighted images often limits the generalisability of radiomics in a multi-center context, texture analysis of both conventional and novel quantitative mapping techniques that have already been studied in PCa^72^ is an area of interest. Finally, whilst this study focused on baseline prediction of PCa progression on AS, there is scope for improving mpMRI performance for the follow-up monitoring of AS patients. With the recently developed Prostate Cancer Radiological Estimation of Change in Sequential Evaluation (PRECISE) scoring system including only subjective criteria for the assessment of radiological progression of the disease^73,74^, the use of delta-radiomics in follow-up mpMRI scans could complement the developed baseline predictive model by acting as a quantitative tool for dynamic re-evaluation of the risk of PCa progression, further increasing confidence in mpMRI as an alternative to repeat biopsies. In this study, we already used PRECISE 5 as an alternative to histopathological progression of the disease, which aligns with the global trend towards heavier reliance on MRI in navigating follow-up in AS and makes the results more applicable to the routine clinical practice where the two surrogates are used interchangeably^52,75–77^.

In conclusion, a combination of clinicopathological predictors and T2WI-derived radiomic features may add benefit in predicting risk of PCa progression on AS. These results pave the way for larger future studies investigating the added value of MRI-derived radiomics to standard-of-care clinical biomarkers for improving baseline risk-stratification of patients suitable for enrollment on AS programmes.

## Supplementary Information

Supplementary Information.

## Data Availability

The primary research data will be made available on Mendeley Data upon the publication of this manuscript.
