# Supplementary material for: MRI-derived radiomics model for baseline prediction of prostate cancer progression on active surveillance
Source: Sci Rep. 2021 Jun 21;11:12917. doi: 10.1038/s41598-021-92341-6 (PMC8217549; doi:10.1038/s41598-021-92341-6)
Supplement: Supplementary file 1 — Supplementary Information. [file 41598_2021_92341_MOESM1_ESM.pdf]

MRI-derived radiomics model for baseline prediction of prostate cancer progression on active surveillance: a proof-of-concept study

Nikita Sushentsev[1†*], Leonardo Rundo[1,2†], Oleg Blyuss[3,4,5], Vincent J Gnanapragasam[6,7], Evis Sala[1,2] Tristan Barrett[1]

Supplementary information

List of abbreviations used:

FSCR	Fisher score
Gini	Gini index
GLMnet	General linear model
KNN	k-nearest neighbours
LDA	Linear discrimination analysis
LG	Logistic regression
MIM	Multivariate mutual information maximization
MRMR	Minimum redundancy maximum relevance
RF	Random forest
SVM	Support vector machine
TSCR	T-score
WLCX	Wilcoxon Score

Supplementary methods.

Radiomic feature extraction.

Due to the small sample size, we chose to use only original features to avoid the processing of additional features extracted using convolutional image filters on the input medical images (e.g. Laplacian of Gaussian, logarithm, exponential, gradient, wavelets). Moreover, clear guidelines for filtered versions of the images are not yet available, with the release of the IBSI Chapter 2 still being under preparation[1]. An established, practical rule states that at least 10 samples (i.e. patients) are needed for each feature in a model based on binary classifiers[2].

References:

1. IBSI 2 – IBSI – Image Biomarker Standardisation Initiative. https://theibsi.github.io/ibsi2/.
2. Gillies, R. J., Kinahan, P. E. & Hricak, H. Radiomics: Images are more than pictures, they are data. *Radiology* **278**, 563–577 (2016).

#	Radiomic feature
First-order	
1	10th Percentile
2	90th Percentile
3	Energy
4	Entropy
5	Interquartile Range
6	Kurtosis
7	Maximum
8	Mean Absolute Deviation
9	Mean
10	Median
11	Minimum
12	Range
13	Robust Mean Absolute Deviation
14	Root Mean Squared
15	Skewness
16	Total Energy
17	Uniformity
18	Variance

Shape-based (3D)	
19	Mesh Volume
20	Voxel Volume
21	Surface Area
22	Surface Area to Volume ratio
23	Sphericity
24	Maximum 3D diameter
25	Maximum 2D diameter (Slice)
26	Maximum 2D diameter (Column)
27	Maximum 2D diameter (Row)
28	Major Axis Length
29	Minor Axis Length
30	Least Axis Length
31	Elongation
32	Flatness

Grey Level Co-occurrence Matrix (GLCM)	
33	Autocorrelation
34	Cluster Prominence
35	Cluster Shade
36	Cluster Tendency
37	Contrast
38	Correlation
39	Difference Average
40	Difference Entropy
41	Difference Variance
42	ID: Inverse Difference
43	IDM: Inverse Difference Moment
44	IDMN: Inverse Difference Moment Normalised
45	IDN: Inverse Difference Normalised
46	IMC 1: Informational Measure of Correlation 1
47	IMC 2: Informational Measure of Correlation 2
48	Inverse Variance
49	Joint Average
50	Joint Energy
51	Joint Entropy
52	MCC: Maximal Correlation Coefficient
53	Maximum Probability
54	Sum Average
55	Sum Entropy
56	Sum Squares

Grey Level Dependence Matrix (GLDM)	
57	Dependence Entropy
58	Dependence NonUniformity
59	Dependence NonUniformity Normalised
60	Dependence Variance
61	Grey Level NonUniformity
62	Grey Level Variance
63	High Grey Level Emphasis
64	Large Dependence Emphasis
65	Large Dependence High Grey Level Emphasis
66	Large Dependence Low Grey Level Emphasis
67	Low Grey Level Emphasis
68	Small Dependence Emphasis
69	Small Dependence High Grey Level Emphasis
70	Small Dependence Low Grey Level Emphasis
Grey Level Run Length Matrix (GLRLM)	
71	Grey Level NonUniformity
72	Grey Level NonUniformity Normalised
73	Grey Level Variance
74	High Grey Level Run Emphasis
75	Long Run Emphasis
76	Long Run High Grey Level Emphasis
77	Long Run Low Grey Level Emphasis
78	Low Grey Level Run Emphasis
79	Run Entropy
80	Run Length NonUniformity
81	Run Length NonUniformity Normalised
82	Run Percentage
83	Run Variance
84	Short Run Emphasis
85	Short Run High Grey Level Emphasis
86	Short Run Low Grey Level Emphasis

Grey Level Size Zone Matrix (GLSZM)	
87	Grey Level NonUniformity
88	Grey Level NonUniformity Normalised
89	Grey Level Variance
90	High Grey Level Zone Emphasis
91	Large Area Emphasis
92	Large Area High Grey Level Emphasis
93	Large Area Low Grey Level Emphasis
94	Low Grey Level Zone Emphasis
95	Size Zone NonUniformity
96	Size Zone NonUniformity Normalised
97	Small Area Emphasis
98	Small Area High Grey Level Emphasis
99	Small Area Low Grey Level Emphasis
100	Zone Entropy
101	Zone Percentage
102	Zone Variance
Neighbouring Grey-Tone Difference Matrix (NGTDM)	
103	Busyness
104	Coarseness
105	Complexity
106	Contrast
107	Strength

Supplementary Table S1. Radiomic features extracted from the ROIs in this study. All radiomic features were extracted using PyRadiomics, and the radiomic feature formulation can be found on the online PyRadiomics documentation (https://pyradiomics.readthedocs.io/en/latest/).

	FSCR	TSCR	WLCX	Gini	MIM	MRMR
KNN	0.591 (0.458-0.724)	0.612 (0.481-0.743)	0.591 (0.458-0.724)	0.591 (0.458-0.724)	0.591 (0.458-0.724)	0.531 (0.397-0.665)
LG	0.609 (0.476-0.742)	0.58 (0.444-0.715)	0.609 (0.476-0.742)	0.609 (0.476-0.742)	0.609 (0.476-0.742)	0.546 (0.409-0.683)
LDA	0.589 (0.455-0.722)	0.557 (0.421-0.694)	0.589 (0.455-0.722)	0.589 (0.455-0.722)	0.589 (0.455-0.722)	0.541 (0.404-0.677)
GLMnet	0.605 (0.473-0.736)	0.584 (0.449-0.719)	0.605 (0.473-0.736)	0.605 (0.473-0.736)	0.605 (0.473-0.736)	0.536 (0.401-0.672)
SVM	0.561 (0.425-0.697)	0.559 (0.425-0.694)	0.561 (0.425-0.697)	0.561 (0.425-0.697)	0.561 (0.425-0.697)	0.444 (0.31-0.579)
RF	0.482 (0.347-0.617)	0.54 (0.406-0.674)	0.482 (0.347-0.617)	0.482 (0.347-0.617)	0.482 (0.347-0.617)	0.474 (0.338-0.61)

Supplementary Table S2. Summary areas under the ROC curve (AUCs) with 95% confidence intervals of predictive models including clinicopathological predictors alone developed using various feature selection and machine learning classification algorithms.

	FSCR	TSCR	WLCX	Gini	MIM	MRMR
KNN	0.569 (0.436-0.702)	0.555 (0.422-0.689)	0.567 (0.434-0.7)	0.535 (0.401-0.669)	0.625 (0.494-0.756)	0.54 (0.407-0.673)
LG	0.565 (0.429-0.701)	0.557 (0.421-0.694)	0.54 (0.403-0.676)	0.55 (0.415-0.685)	0.563 (0.43-0.697)	0.488 (0.353-0.623)
LDA	0.565 (0.43-0.701)	0.56 (0.424-0.697)	0.529 (0.393-0.665)	0.56 (0.426-0.695)	0.573 (0.44-0.706)	0.498 (0.363-0.634)
GLMnet	0.374 (0.244-0.505)	0.165 (0.068-0.261)	0.595 (0.462-0.727)	0.353 (0.224-0.481)	0.583 (0.45-0.715)	N/A
SVM	0.155 (0.055-0.255)	0.235 (0.118-0.353)	0.478 (0.343-0.614)	0.417 (0.281-0.552)	0.485 (0.349-0.621)	0.224 (0.115-0.333)
RF	0.617 (0.485-0.748)	0.59 (0.457-0.723)	0.515 (0.379-0.65)	0.59 (0.456-0.724)	0.565 (0.429-0.7)	0.531 (0.396-0.665)

Supplementary Table S3. Summary AUCs with 95% confidence intervals of predictive models including T2WI-derived radiomic features alone.

	FSCR	TSCR	WLCX	Gini	MIM	MRMR
KNN	0.624 (0.493-0.755)	0.391 (0.261-0.522)	0.643 (0.513-0.773)	0.592 (0.46-0.723)	0.55 (0.418-0.682)	0.544 (0.409-0.68)
LG	0.544 (0.41-0.679)	0.434 (0.3-0.568)	0.55 (0.415-0.684)	0.568 (0.435-0.702)	0.525 (0.39-0.66)	0.58 (0.445-0.714)
LDA	0.545 (0.411-0.68)	0.446 (0.312-0.58)	0.55 (0.415-0.684)	0.571 (0.437-0.704)	0.538 (0.403-0.673)	0.602 (0.468-0.736)
GLMnet	0.637 (0.507-0.767)	0.408 (0.273-0.542)	0.623 (0.491-0.754)	0.607 (0.474-0.74)	0.614 (0.483-0.744)	0.541 (0.405-0.676)
SVM	0.428 (0.295-0.561)	0.402 (0.268-0.535)	0.547 (0.412-0.681)	0.511 (0.375-0.647)	0.468 (0.332-0.605)	0.593 (0.46-0.726)
RF	0.582 (0.447-0.716)	0.334 (0.207-0.461)	0.648 (0.518-0.777)	0.652 (0.525-0.779)	0.631 (0.499-0.764)	0.535 (0.399-0.67)

Supplementary Table S4. Summary AUCs with 95% confidence intervals of predictive models including ADC-derived radiomic features alone.

	FSCR	TSCR	WLCX	Gini	MIM	MRMR
KNN	0.573 (0.434-0.711)	0.568 (0.437-0.699)	0.628 (0.5-0.756)	0.565 (0.434-0.696)	0.475 (0.341-0.61)	0.554 (0.42-0.687)
LG	0.57 (0.437-0.703)	0.547 (0.411-0.684)	0.555 (0.421-0.688)	0.511 (0.377-0.646)	0.481 (0.345-0.618)	0.601 (0.468-0.733)
LDA	0.562 (0.428-0.695)	0.56 (0.425-0.696)	0.503 (0.368-0.638)	0.511 (0.375-0.646)	0.509 (0.373-0.645)	0.629 (0.498-0.759)
GLMnet	0.64 (0.511-0.769)	0.586 (0.453-0.718)	0.639 (0.51-0.768)	0.539 (0.403-0.675)	0.563 (0.428-0.698)	0.351 (0.224-0.479)
SVM	0.529 (0.394-0.663)	0.489 (0.353-0.624)	0.489 (0.354-0.625)	0.335 (0.205-0.466)	0.296 (0.167-0.425)	0.282 (0.159-0.404)
RF	0.601 (0.469-0.733)	0.595 (0.461-0.729)	0.549 (0.414-0.684)	0.549 (0.414-0.684)	0.576 (0.443-0.709)	0.519 (0.383-0.655)

Supplementary Table S5. Summary AUCs with 95% confidence intervals of predictive models including a combination of clinicopathological predictors, T2WI-, and ADC-derived radiomic features.

	FSCR	TSCR	WLCX	Gini	MIM	MRMR
KNN	0.583 (0.45-0.715)	0.606 (0.476-0.735)	0.749 (0.636-0.862)	0.548 (0.414-0.683)	0.615 (0.486-0.744)	0.621 (0.49-0.752)
LG	0.547 (0.409-0.686)	0.625 (0.493-0.757)	0.599 (0.464-0.734)	0.546 (0.411-0.681)	0.584 (0.45-0.718)	0.565 (0.429-0.7)
LDA	0.558 (0.42-0.696)	0.612 (0.478-0.746)	0.579 (0.442-0.715)	0.547 (0.41-0.683)	0.606 (0.473-0.739)	0.51 (0.374-0.646)
GLMnet	0.622 (0.492-0.752)	0.55 (0.415-0.686)	0.652 (0.523-0.78)	0.591 (0.458-0.724)	0.608 (0.476-0.74)	0.379 (0.248-0.51)
SVM	0.443 (0.309-0.577)	0.41 (0.278-0.542)	0.647 (0.514-0.779)	0.508 (0.373-0.642)	0.586 (0.453-0.72)	0.309 (0.185-0.433)
RF	0.606 (0.473-0.738)	0.6 (0.466-0.733)	0.598 (0.466-0.73)	0.597 (0.463-0.73)	0.618 (0.488-0.749)	0.54 (0.405-0.675)

Supplementary Table S6. Summary AUCs with 95% confidence intervals of predictive models including a combination of clinicopathological predictors and T2WI-derived radiomic features.

	FSCR	TSCR	WLCX	Gini	MIM	MRMR
KNN	0.568 (0.434-0.702)	0.512 (0.377-0.646)	0.635 (0.505-0.765)	0.634 (0.504-0.764)	0.615 (0.483-0.746)	0.578 (0.445-0.71)
LG	0.619 (0.488-0.75)	0.492 (0.357-0.627)	0.595 (0.462-0.729)	0.547 (0.412-0.683)	0.618 (0.487-0.749)	0.632 (0.503-0.761)
LDA	0.601 (0.469-0.733)	0.453 (0.318-0.587)	0.545 (0.41-0.68)	0.579 (0.444-0.713)	0.584 (0.452-0.716)	0.64 (0.512-0.768)
GLMnet	0.67 (0.544-0.796)	0.575 (0.442-0.708)	0.663 (0.535-0.791)	0.611 (0.478-0.743)	0.671 (0.546-0.797)	0.574 (0.441-0.706)
SVM	0.609 (0.476-0.742)	0.278 (0.162-0.394)	0.615 (0.483-0.747)	0.508 (0.374-0.643)	0.652 (0.523-0.781)	0.521 (0.386-0.656)
RF	0.577 (0.44-0.714)	0.507 (0.372-0.642)	0.602 (0.467-0.738)	0.598 (0.465-0.73)	0.606 (0.472-0.74)	0.555 (0.417-0.693)

Supplementary Table S7. Summary AUCs with 95% confidence intervals of predictive models including a combination of clinicopathological predictors and ADC-derived radiomic features.

	FSCR	TSCR	WLCX	Gini	MIM	MRMR
KNN	0.516 (0.381-0.652)	0.489 (0.353-0.625)	0.559 (0.425-0.693)	0.565 (0.434-0.696)	0.475 (0.341-0.61)	0.466 (0.332-0.6)
LG	0.487 (0.351-0.623)	0.512 (0.377-0.648)	0.406 (0.274-0.538)	0.511 (0.377-0.646)	0.481 (0.345-0.618)	0.617 (0.484-0.749)
LDA	0.477 (0.341-0.612)	0.514 (0.378-0.649)	0.413 (0.28-0.545)	0.511 (0.375-0.646)	0.509 (0.373-0.645)	0.595 (0.462-0.729)
GLMnet	0.588 (0.454-0.722)	0.389 (0.256-0.523)	0.591 (0.457-0.725)	0.539 (0.403-0.675)	0.563 (0.428-0.698)	N/A
SVM	0.312 (0.186-0.438)	0.31 (0.18-0.44)	0.288 (0.164-0.412)	0.335 (0.205-0.466)	0.296 (0.167-0.425)	0.198 (0.094-0.301)
RF	0.562 (0.426-0.698)	0.537 (0.401-0.672)	0.537 (0.403-0.671)	0.549 (0.414-0.684)	0.576 (0.443-0.709)	0.498 (0.362-0.633)

Supplementary Table S8. Summary AUCs with 95% confidence intervals of predictive models including a combination of T2WI- and ADC-derived radiomic features.

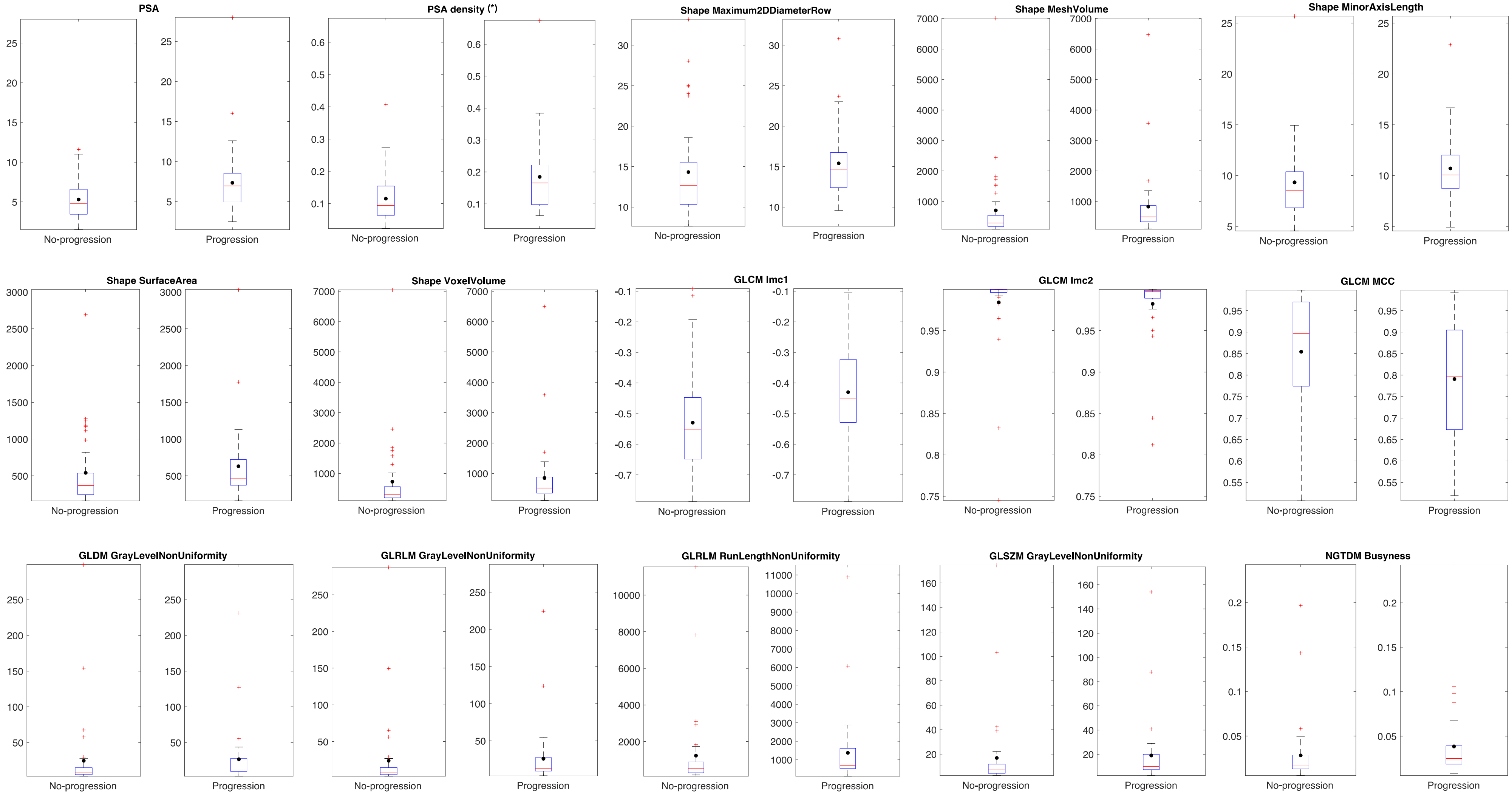

Supplementary Figure S1. Boxplots of the 15 features selected by the best performing model. For each feature, a non-parametric Wilcoxon rank sum test (Mann-Whitney U test) was performed by sub-dividing the patients into non-progressors and progressors (significance level set to 0.05). The p-values were adjusted using the Bonferroni-Holm method for multiple comparison tests. * $p < 0.05$.
